# Determinants of Recovery From Obstetric Fistula in Ethiopia: A Systematic Review and Meta‐Analysis

**DOI:** 10.1002/nop2.70680

**Published:** 2026-07-06

**Authors:** Chalie Mulugeta, Tadele Emagneneh, Assefa Sisay, Getinet Kumie, Esuyawkal Mislu, Henok Kumsa, Adem Yesuf, Abebaw Alamrew

**Affiliations:** ^1^ Department of Midwifery, College of Health Science Woldia University Weldiya Ethiopia; ^2^ Department of Medical Laboratory, College of Health Science Woldia University Weldiya Ethiopia

**Keywords:** associated factors, Ethiopia, obstetric fistula, recovery, time to recovery

## Abstract

**Aim:**

This study aimed to assess the prevalence of recovery from obstetric fistula and identify factors associated with recovery amongst women in Ethiopia.

**Design:**

A systematic review and meta‐analysis of retrospective cohort studies were conducted.

**Methods:**

A comprehensive search was performed in PubMed, Google Scholar, EMBASE, Cochrane Library and Hinari using relevant keywords and Boolean operators. In addition, grey literature sources such as Google Scholar, institutional repositories and reports from international organisations. Data extraction was done using Microsoft Excel, and statistical analyses were conducted in Stata version 11. The methodological quality of the studies was assessed using the Newcastle–Ottawa Scale. Publication bias was evaluated with a funnel plot and Egger's test, and heterogeneity was measured using the I‐squared statistic.

**Results:**

Ten studies including 2382 participants from four regions of Ethiopia, were analysed. The pooled prevalence of recovery from obstetric fistula was 80.42% (95% CI: 75.74–85.10). Factors significantly associated with recovery included: maternal height ≤ 150 cm (AHR: 1.30; 95% CI: 1.14–1.47; *I* ^2^ = 83.5%), antenatal care attendance (AHR: 1.30; 95% CI: 1.08–1.52; *I* ^2^ = 96.8%), institutional delivery (AHR: 1.40; 95% CI: 1.12–1.70; *I* ^2^ = 97.3%), duration of incontinence < 3 months (AHR: 1.41; 95% CI: 1.11–1.71), intact urethra (AHR: 2.27; 95% CI: 1.39–3.14; *I* ^2^ = 99.3%), vaginal delivery (AHR: 1.69; 95% CI: 1.10–2.28; *I* ^2^ = 99.1%) and fistula width ≤ 2 cm (AHR: 1.43; 95% CI: 1.05–1.81; *I* ^2^ = 97%).

**No Patient or Public Contribution:**

This study was based on previously published studies, and no direct patient or public involvement was included.

**Conclusion:**

Recovery from obstetric fistula in Ethiopia remains suboptimal. Factors significantly associated with better recovery outcomes included maternal height, antenatal care attendance, institutional delivery, mode of delivery, duration of incontinence ≤ 3 months, intact urethra and fistula width ≤ 2 cm. Targeted interventions focusing on these factors—such as increasing antenatal care uptake, promoting institutional deliveries and ensuring early management of incontinence—are essential to improve recovery outcomes for affected women.

**Trial Registration:** PROSPERO: CRD420261422331

## 1 Background

Obstetric fistula is a severe maternal health condition characterised by an abnormal opening between the vagina and the bladder and/or rectum, resulting in continuous leakage of urine and/or faeces (De Bernis 2007). The World Health Organization defines it as a childbirth injury most commonly caused by prolonged, obstructed labour, during which sustained pressure from the foetal head damages the mother's soft tissues and forms a fistula (UNFPA 2012). Obstructed labour not only contributes to maternal morbidity and mortality but also serves as the principal pathway leading to obstetric fistula (Lewis and De Bernis 2006).

Globally, an estimated 2–3.5 million women in developing countries live with obstetric fistula, with 50,000–100,000 new cases occurring each year (UNFPA 2012). Over 2 million young women in Asia and sub‐Saharan Africa remain untreated (Sheet 2018; Region WiA 2018). In Ethiopia, approximately 142,000 women live with untreated fistulas, with 9000 new cases annually, yet only 1200 receive treatment (Ethiopia HAHFPI 2023). Women affected by obstetric fistula experience severe physical, social and psychological consequences, including incontinence, social isolation, stigma and reduced quality of life (Lewis and De Bernis 2006).

About 800 women die from pregnancy or childbirth‐related complications around the world every day. Maternal morbidity is estimated to affect at least 20 women for every woman who dies of reasons connected to pregnancy; obstetric fistula is one of the most severe types of this condition (UNFPA 2012). However, it is believed that more than 90% of fistulas in underdeveloped nations are obstetric in nature. In comparison, more than 70% in the United States and the United Kingdom are the result of pelvic surgery (Cook et al. 2004). Incidence rates in low‐ and middle‐income countries vary widely, with estimates ranging from 0 to 4.09 cases per 1000 deliveries and prevalence estimates from 0 to 81 cases per 1000 women (Cowgill et al. 2015). A meta‐analysis in sub‐Saharan Africa found a lifetime prevalence of 3 per 1000 reproductive‐age women, with Ethiopia reporting the majority of cases (Maheu‐Giroux et al. 2015).

The underlying reasons for fistula in developing countries include a mix of low women's status, poverty, illiteracy and a lack of access to effective emergency obstetric treatment (Capes et al. 2011). If prompt, effective medical care is not offered, obstetric fistulas can have serious physical, social and psychological consequences. It results in either urine, faecal or both forms of incontinence (Ahmed and Holtz 2007). If left untreated, fistulas lead to ongoing urinary or faecal incontinence and substantial psychosocial consequences, including stigma, discrimination and emotional distress (Nduka et al. 2023; Wall et al. 2005).

Surgical repair remains the mainstay of treatment for obstetric fistula, with the primary goal of restoring urinary continence and normal pelvic function. The success of surgical repair is influenced by several factors, including fistula characteristics, the surgeon's expertise and the surgical technique employed (Wall et al. 2001; Frajzyngier, Ruminjo, Asiimwe, et al. 2012; Mselle et al. 2011; Frajzyngier, Ruminjo, and Barone 2012). Preventive measures, including adequate prenatal care and timely caesarean delivery, are critical to avoid recurrence (Pope 2018).

Previous studies have identified multiple factors associated with successful recovery from obstetric fistula. These include socio‐demographic, obstetric and clinical variables such as maternal education, parity, duration of labour, fistula size, urethral status, place and mode of delivery, marital status, residence, age, anthropometric characteristics, ANC follow‐up and duration of incontinence (Zeleke et al. 2024; Ejigu et al. 2023; Hareru et al. 2024; Egziabher et al. 2015; Kapaya 2019; Bihon 2022; Yismaw et al. 2019; Ambese et al. 2023; Areba et al. 2022; Hussen and Melese 2017; Bihon and Meikena 2022).

Despite these findings, existing studies in Ethiopia are often limited by regional variability, small sample sizes and lack of national representativeness. Therefore, this study aimed to estimate the pooled prevalence of recovery from obstetric fistula and to identify its associated factors at the national level. By providing a comprehensive synthesis of available evidence, this study offers important insights for identifying gaps in healthcare services and informing policy and programme development to improve maternal health outcomes and recovery rates amongst affected women.

## 2 Methods

### 2.1 Study Protocol and Reporting

The present study was conducted in accordance with the Preferred Reporting Items for Systematic Reviews and Meta‐Analyses (PRISMA) guideline (Page et al. 2021). The eligibility criteria were adapted from the Newcastle–Ottawa Scale (NOS) 2016 review guidelines (Downes 2016). The completed PRISMA 2020 checklist is provided as File S1. This systematic review and meta‐analysis was retrospectively registered in PROSPERO (registration number: CRD420261422331; registered on 13 June 2026; https://www.crd.york.ac.uk/PROSPERO/view/CRD420261422331). EndNote (version X7) reference management software was used to download, organise and manage relevant studies, whilst Zotero was used for citation management.

### 2.2 Inclusion Criteria

All quantitative retrospective cohort studies were conducted on recovery from obstetric fistula and associated factors in Ethiopia. Only published articles written in English were included without a time limit.

### 2.3 Exclusion Criteria

The analysis did not include duplicate studies, anonymous reports and articles without an abstract or full text, studies with different outcome interests or qualitative studies. Furthermore, after at least two emails were exchanged with the primary author, studies that did not include outcomes in either the exposed or non‐exposed groups were excluded. Because it was impossible to extract data from these studies in the absence of hard data, they were excluded.

### 2.4 Outcome of Interest

The outcome of interest was recovery from obstetric fistula and contributing factors.

### 2.5 Variables

#### 2.5.1 Time to Recovery (Time‐To‐Event Outcome)

Time to recovery was defined as the number of days from the date of surgical repair of obstetric fistula to the date of hospital discharge with documented recovery. This variable represents a time‐to‐event outcome and was measured in days (Hussen and Melese 2017; Bihon and Meikena 2022).

#### 2.5.2 Recovery (Binary Outcome)

Recovery from obstetric fistula was defined as the clinical condition of being cured following surgical repair, characterised by continence and discharge from hospital as recovered. This outcome was treated as a binary variable (recovered vs. not recovered) and was used to estimate the prevalence of recovery (Bihon and Meikena 2022).

### 2.6 Search Strategy

A comprehensive literature search was conducted from 1 February to 29 February 2025 to identify relevant studies. Electronic databases, including PubMed, HINARI, EMBASE and Cochrane Library, were systematically searched. In addition, grey literature was also explored to minimise publication bias. Grey literature was defined as research not formally published in peer‐reviewed journals, including theses, dissertations, organisational reports and preprints. Sources of grey literature included Google Scholar, institutional repositories and reports from international organisations such as the World Health Organization (WHO) and the United Nations (UN).

The search strategy was developed using a combination of keywords and Boolean operators. Initially, relevant keywords were identified from article titles and abstracts in PubMed, Google and Google Scholar. Subsequently, similar and related terms were identified and refined. The reference lists of included studies were also screened to identify additional relevant articles.

The following keywords and phrases were used: ‘time to recovery’, ‘recovery’, ‘fistula’, ‘obstetric fistula’, ‘time’, ‘childbirth’, ‘maternal health services’, ‘associated factors’, ‘predictors’, ‘determinants’, ‘contributing factors’, ‘prevalence’, ‘magnitude’, ‘proportion’ and ‘Ethiopia’. Combined search terms such as ‘time to recovery AND obstetric fistula’ were also applied. Boolean operators (AND, OR) were used to combine different concepts and refine the search strategy through iterative testing.

The search was conducted across all databases and grey literature sources without restriction on publication status, and appropriate time limits were applied as per the study protocol. The inclusion of grey literature helped to reduce publication bias by capturing unpublished and non‐peer‐reviewed studies that may not be indexed in major databases.

### 2.7 Data Extraction

The data were extracted using Microsoft Excel. Two distinct data extraction formats were used to collect information for analysis. For the prevalence outcome, the following variables were extracted: author's last name, year of publication, study location, study design, sample size, number of women with recovery from obstetric fistula, prevalence estimates with 95% confidence intervals and study quality score.

For contributing factors, adjusted hazard ratios (HRs) with their corresponding 95% confidence intervals were extracted for all associated factors from the included studies. Since all studies reported effect estimates as hazard ratios, no transformation of effect measures was required. When multiple adjusted models were presented, the most fully adjusted model was selected to minimise confounding bias. For meta‐analysis, hazard ratios were assumed to be comparable across studies despite differences in covariate adjustment. To address variability in study definitions, similar variables were grouped into standardised categories based on clinical and epidemiological similarity. A random‐effects model was applied to account for between‐study heterogeneity.

The author's last name and year of publication were also included in the data extraction format for contributing factors.

### 2.8 Quality Assessment/Critical Appraisal

The methodological quality of the included studies was assessed using the Newcastle–Ottawa Scale (NOS) for cohort studies (Table S1) (Downes 2016). The NOS evaluates observational studies across three domains: selection of study participants, comparability of study groups and outcome assessment. The selection domain (4 points) assesses representativeness of the exposed cohort, selection of the non‐exposed cohort, ascertainment of exposure and confirmation that the outcome of interest was not present at baseline. The comparability domain (2 points) evaluates adjustment for confounding factors, whilst the outcome domain (3 points) assesses outcome measurement, adequacy of follow‐up and completeness of follow‐up.

Each study was scored on a scale of 0 to 9. Studies scoring ≥ 6 were considered to have low risk of bias (high quality), whilst those scoring < 6 were considered high risk of bias. Quality assessment was independently conducted by two reviewers (AA and TE). Any disagreements were resolved through discussion and consensus, and when necessary, a third reviewer (EM) was consulted to reach a final decision. The results of the quality assessment were used for the interpretation of findings and sensitivity analyses.

### 2.9 Study Selection

Study selection was performed independently by two reviewers based on predefined inclusion and exclusion criteria. Titles and abstracts were screened first, followed by full‐text review. Disagreements between reviewers were resolved through discussion and consensus, and when necessary, a third reviewer (AY) was consulted to make the final decision. Eligible studies were included in the final analysis.

### 2.10 Statistical Analysis

Stata statistical software version 11.0 was used to perform all analyses. A random‐effects meta‐analysis model, based on the DerSimonian and Laird method, was used to pool the prevalence of recovery from obstetric fistula in Ethiopia. A 95% confidence interval and *p*‐value were used to determine statistical significance. A random‐effects model was applied in the presence of significant heterogeneity. Statistical heterogeneity was assessed using the *I* ^2^ statistic. Subgroup analyses were conducted to explore potential sources of heterogeneity (Migliavaca et al. 2022).

Meta‐regression analyses were performed to examine the effect of study‐level covariates, including sample size and year of publication, on the pooled prevalence. Restricted maximum likelihood (REML) was used to estimate between‐study variance.

Publication bias was assessed using visual inspection of funnel plots and Egger's regression test. Egger's test indicated potential publication bias (*p* = 0.03) (Egger et al. 1997). The *p*‐value was < 0.05; there was statistical evidence for the presence of publication bias using Egger's test was 0.03. In addition, the trim‐and‐fill method was used to estimate the potential impact of unpublished studies on the pooled estimate. A leave‐one‐out sensitivity analysis was performed to assess the robustness of the pooled prevalence estimate.

The meta‐analysis was conducted under the supervision of a researcher with advanced statistical expertise.

## 3 Results

### 3.1 Searching Findings

A total of 950 studies (PubMed = 100, Hinari = 10, Cochrane Review = 100, EMBASE = 20 and Google Scholar = 720) published were identified. Of all the articles, 200 were removed because they were duplicates. Based on the inclusion and exclusion criteria of title and abstract selection, the eligibility of 750 abstracts was evaluated. The articles that did not fulfil the criteria were removed (*n* = 690), leaving a total of 60 articles for full‐text screening.

**FIGURE 1:**
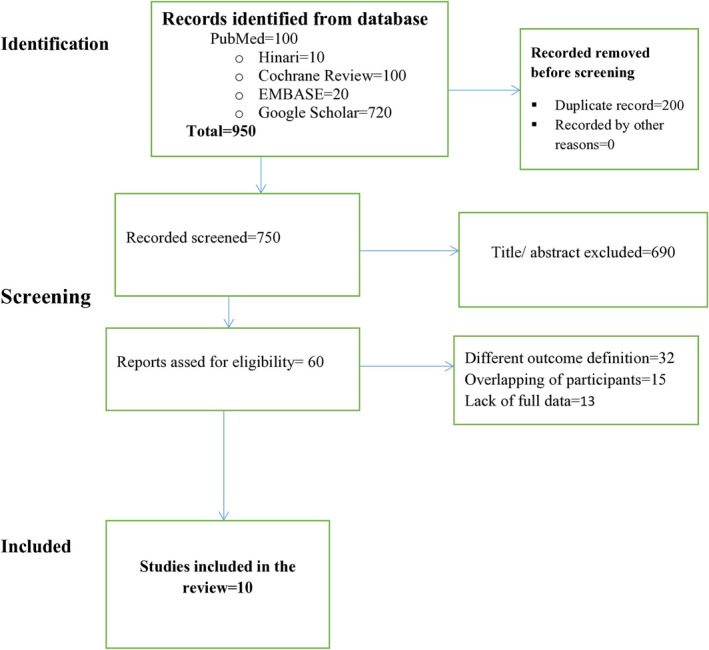
PRISMA flow diagram illustrating the study identification, screening, eligibility assessment and inclusion process for the systematic review and meta‐analysis on recovery from obstetric fistula.

Sixty articles were screened according to the eligibility criteria for full‐text selection. Furthermore, 50 articles were excluded because the researchers reported different outcomes of interest, overlapping study participants, poor methodological quality or a lack of complete data. Finally, 10 studies were included in the meta‐analysis. The same data source was used to prevent participant overlap, and one or more publications were eliminated based on the studies' overall quality scores, with the highest‐scoring research being included (Figure 1).

### 3.2 Study Characteristics of Recovery From Obstetric Fistula

A total of 10 studies were included in our analysis, encompassing 3238 women, of whom 2382 recovered from obstetric fistula. All included studies were published articles and employed a retrospective cohort design reporting time to recovery from obstetric fistula. Sample sizes across the studies ranged from 206 (Temesgen 2016) to 612 (Yismaw et al. 2019) (Table 1).

**TABLE 1:** Description of studies included in this systematic review and meta‐analysis on prevalence and factors of time to recovery from obstetric fistula in Ethiopia, 2024.

Id no	Author	Year	Study design	Region	Sample size	Frequency	Effect size [95% C]
1	Getachew (2015)	2015	Retrospective cohort	Sidama	360	294	81.7 (77.7–85.69)
2	Ambese et al. (2023)	2022	Retrospective cohort	Tigray	224	181	80.8 (75.64–85.96)
3	Derso et al. (2020)	2020	Retrospective cohort	Amhara	289	—	—
4	Yismaw et al. (2019)	2019	Retrospective cohort	Amhara	612	539	88.07 (85.5–90.64)
5	Hussen and Melese (2017)	2017	Retrospective cohort	Oromia	433	291	67.21 (62.8–71.6)
6	Areba et al. (2022)	2022	Retrospective cohort	Oromia	270	220	81.4 (76.76–86.04)
7	Bihon and Meikena (2022)	2022	Retrospective cohort	Tigray	328	293	89.3 (85.99–92.67)
8	Kemal (2015)	2015	Retrospective cohort	Oromia	246	187	76.2 (70.8–81.5)
9	Demissie (2018)	2017	Retrospective cohort	Oromia	270	220	81.48 (76.8–86.1)
10	Temesgen (2016)	2016	Retrospective cohort	Oromia	206	157	76.2 (70.4–82.02)

### 3.3 Pooled Recovery Rate From Obstetric Fistula in Ethiopia

**FIGURE 2:**
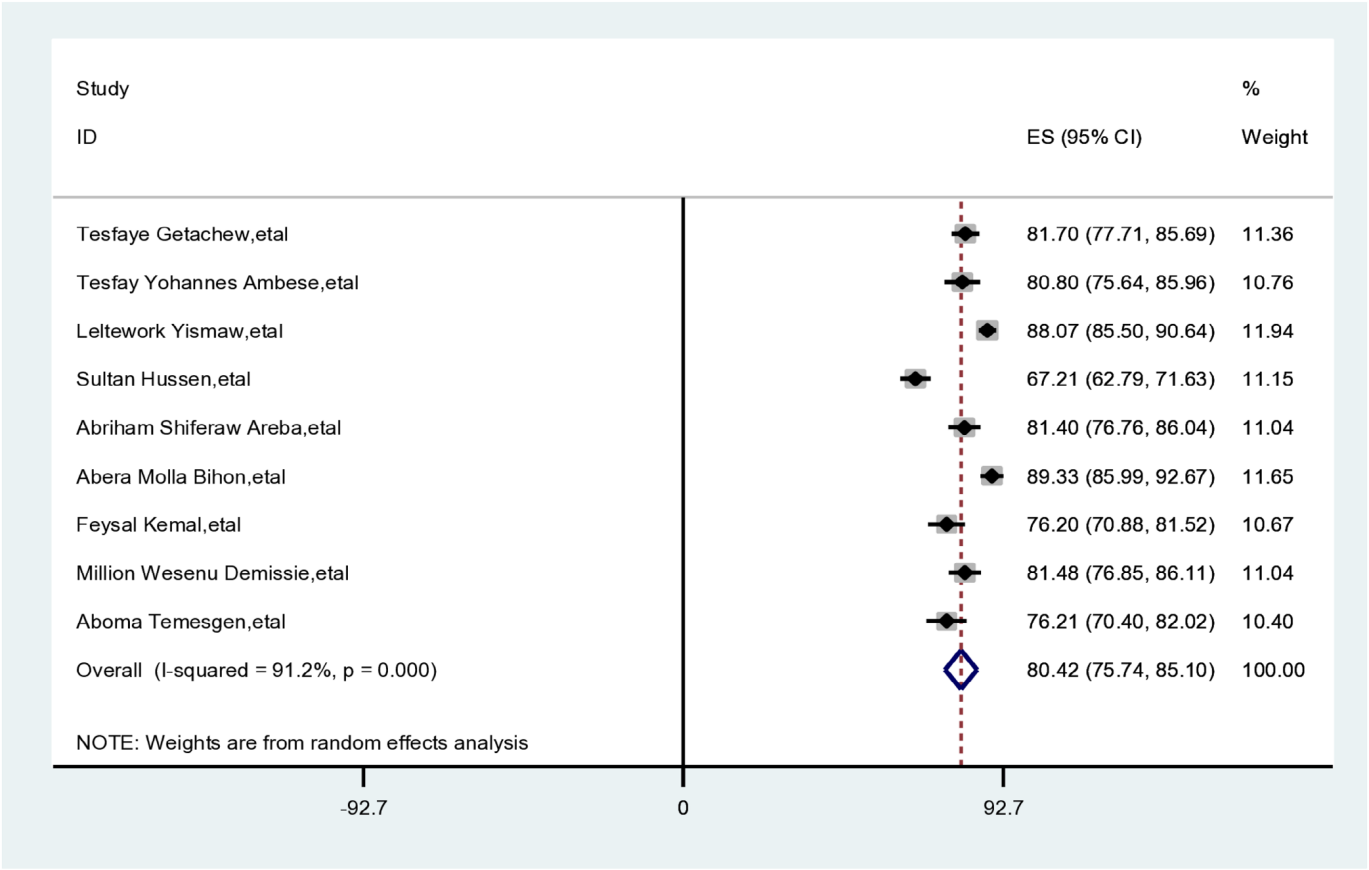
Forest plot displaying the pooled prevalence of recovery from obstetric fistula in Ethiopia.

This meta‐analysis shows the pooled prevalence of recovery from obstetric fistula in Ethiopia. Individual study estimates (ES) and their 95% confidence intervals (CI) are shown, along with their respective weights in the random‐effects meta‐analysis. The overall pooled prevalence of recovery from obstetric fistula is 80.42% (95% CI: 75.74–85.10) (Figure 2). Using the random effects model, the pooled effect size of time to recovery from obstetric fistula showed significant heterogeneity between the included studies (*I* ^2^) = 91.2%, Eggertest0.030 (*p* < 0.001).

### 3.4 Subgroup Analysis of Recovery From Obstetric Fistula

Subgroup analyses were conducted to explore potential sources of heterogeneity and to examine the influence of publication year and geographic region on the reported recovery rates from obstetric fistula.

#### 3.4.1 Analysis by Publication Year

The pooled recovery rate varied by the year of publication. The estimate was lowest for studies published in 2017 (74.32%) and highest for the single study published in 2019 (88.07%). The considerable heterogeneity (*I* ^2^ > 50%) observed within most year subgroups suggests significant variation amongst studies published in the same year.

#### 3.4.2 Analysis by Geographic Region

Recovery rates also differed across the geographic regions of Ethiopia, where the studies were conducted. The highest recovery rate was reported in the Amhara region (88.07%), based on a single study, followed by the Tigray region (85.3%). Studies conducted in the Oromia region reported a lower pooled recovery rate of 76.48%. The significant heterogeneity within the Tigray and Oromia subgroups suggests variations in outcomes between studies even within the same region (Table 2).

**TABLE 2:** Subgroup analysis of obstetric fistula recovery rates by publication year and geographic region in Ethiopia 2024.

Variable	Characteristics	No of study	Pooled prevalence (%)	*I* ^2^	*p*
Year	2015	2	79.243	61.9%	0.105
	2022	3	84.079	82.2%	0.004
	2019	1	88.07	0	0.00
	2017	2	74.32	94.8%	0.00
	2016	1	76.2	—	0.00
Region	Sidama	1	81.7	—	0.00
	Tigray	2	85.3	86.5%	0.007
	Amhara	1	88.07	—	0.00
	Oromia	5	76.48	84.4%	0.00

### 3.5 Publication Bias of Recovery From Obstetric Fistula

**FIGURE 3:**
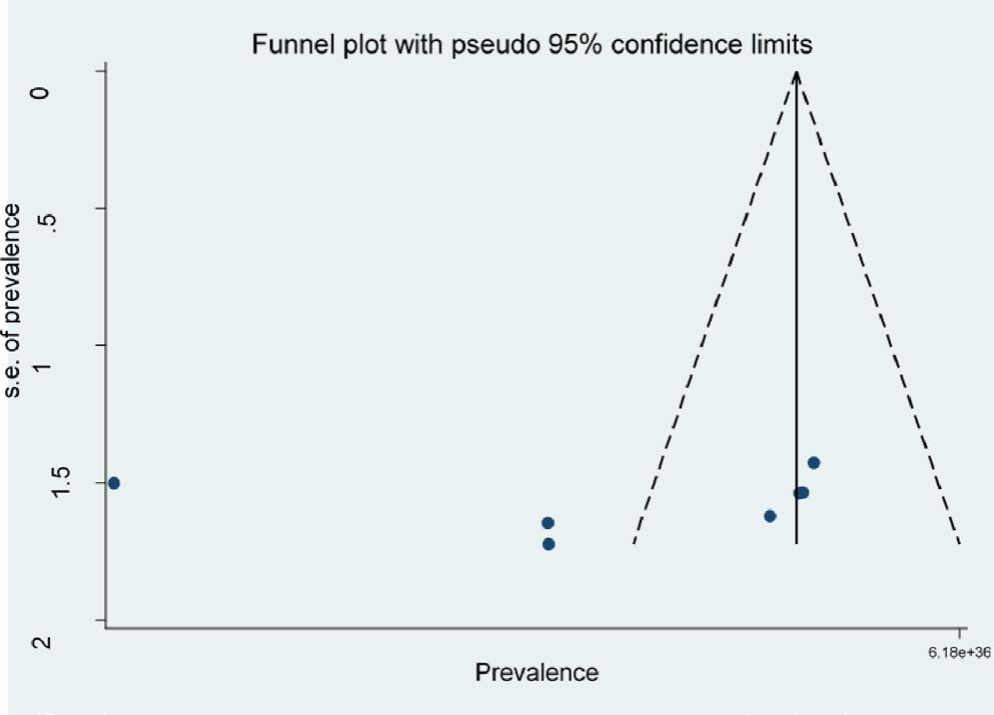
Funnel plot assessing potential publication bias for the systematic review and meta‐analysis on the prevalence of recovery from obstetric fistula in Ethiopia.

Visual inspection of the funnel plot suggested some asymmetry, and Egger's test indicated potential publication bias for the pooled prevalence estimate (*p* = 0.03) (Egger et al. 1997). However, the trim‐and‐fill analysis showed only a minimal change in the pooled estimate after adjustment for potentially missing studies. This apparent discrepancy suggests that although small‐study effects or publication bias may be present, their impact on the overall pooled estimate is limited. Therefore, the main findings remain relatively robust despite evidence of asymmetry, and should be interpreted with appropriate caution (Figure 3).

### 3.6 Heterogeneity of Recovery From Obstetric Fistula

The pooled estimates showed substantial heterogeneity across the included studies. The *I* ^2^ values ranged from 83.5% for maternal height ≤ 150 cm to 99.3% for intact urethra, indicating considerable variability between studies. Such heterogeneity may reflect differences in study populations, regional surgical techniques, surgeon expertise, postoperative care protocols and data collection methods. This high level of heterogeneity suggests that the pooled estimates represent average effects across studies rather than precise or uniform estimates applicable to all settings. Therefore, the findings should be interpreted with caution, as contextual factors may significantly influence recovery outcomes across different settings in Ethiopia.

### 3.7 Meta‐Regression of Recovery From Obstetric Fistula by Sample Size and Year

A meta‐regression was conducted to examine whether study sample size influenced the reported prevalence. The analysis included nine studies (*k* = 9) and used the REML estimator for between‐study variance. Residual heterogeneity was negligible (tau2 0, *I* ^2^ = 0.0%), indicating that the variability in prevalence was fully accounted for by within‐study error. The regression coefficient for sample size was negative but not statistically significant (estimate = −0.0025, SE = 0.0030, *z* = −0.825, *p* = 0.409; 95% CI: −0.0084 to 0.0034), suggesting that sample size did not meaningfully affect prevalence.

A separate meta‐regression was performed to evaluate the effect of publication year. In this model, residual heterogeneity remained substantial (Tau^2^ = 0.1486, *I* ^2^ = 97.88%) and the model explained between‐study variability (*R* ^2^ = 16.35%). The coefficient for year was positive (estimate = 0.0732) but not statistically significant (*z* = 1.593, *p* = 0.111; 95% CI: −0.017 to 0.163), indicating a non‐significant trend towards higher prevalence in more recent studies. Sample size had no detectable impact on reported prevalence, and year of publication showed a non‐significant positive trend. Most variability across studies remains unexplained, highlighting the need to consider additional moderators in future analyses (Table 3).

**TABLE 3:** Meta‐regression of recovery from obstetric fistula by sample size and year.

Moderator	Estimate	SE	*z*‐value	*p*	95% CI (Lower)	95% CI (Upper)	Tau^2^	*I* ^2^	*R* ^2^
Sample size	−0.0025	0.0030	−0.825	0.409	−0.0084	0.0034	0	0.0%	0.0%
Year	0.0732	0.0460	1.593	0.111	−0.017	0.163	0.1486	97.88%	16.35%

### 3.8 Trim‐and‐Fill Analysis for the Prevalence of Recovery From Obstetric Fistula

A Trim and Fill analysis was performed to assess the potential impact of publication bias on the pooled estimate of recovery rates. The results of this analysis are presented in Table 2. The output showed no difference between the original meta‐analysis and the filled analysis. Specifically, the pooled estimates, confidence intervals and number of studies remained identical for both the fixed‐effect (Pooled estimate = 81.339%, 95% CI: 80.384–82.293) and random‐effects models (Pooled estimate = 80.306%, 95% CI: 75.761–84.851) before and after the iterative trimming and filling procedure. As the algorithm imputed zero missing studies, we conclude that there is no statistical evidence of publication bias in the present meta‐analysis, and the overall pooled recovery rate is robust (Table 4).

**TABLE 4:** Trim and fill analysis for the pooled prevalence of recovery from obstetric fistula in Ethiopia.

Method	Pooled estimate (%)	95% CI	Asymmetric *Z* value	*p*	No. of studies
Original Meta‐analysis					
Fixed effect	81.339	80.384 to 82.293	166.981	< 0.001	9
Random effects	80.306	75.761 to 84.851	34.631	< 0.001	9
Filled Meta‐analysis					
Fixed effect	81.339	80.384 to 82.293	166.981	< 0.001	9
Random effects	80.306	75.761 to 84.851	34.631	< 0.001	9

### 3.9 Sensitivity Analysis of Recovery From Obstetric Fistula

A leave‐one‐out sensitivity analysis was performed to evaluate the influence of each individual study on the overall pooled recovery rate. The results, detailed in Table 4, demonstrate that the meta‐analysis is robust. The point estimate for the overall recovery rate remained stable, fluctuating within a narrow range of 79.3% to 82.3% upon the sequential removal of each study. Furthermore, the confidence intervals of all recalculated estimates showed substantial overlap with the original pooled estimate of 80.4% (95% CI: 75.7–85.1). This indicates that no single study exerted undue influence on the overall result, confirming the stability and reliability of the meta‐analysis findings (Table 5).

**TABLE 5:** shows the sensitivity analysis of recovery from obstetric fistula in Ethiopia.

Study omitted	Estimate	95% Conf.	Interval
Tesfaye Getachew et al.	80.234009	74.888924	85.579094
Tesfay Yohannes Ambese et al.	80.36055	75.179802	85.54129
Endeshaw Assefa Derso et al.	80.421326	75.738724	85.103928
Leltework Yismaw et al.	79.385925	74.428398	84.343452
Sultan Hussen et al.	82.262535	78.748764	85.776306
Abriham Shiferaw Areba et al.	80.281326	75.037224	85.525429
Abera Molla Bihon et al.	79.25032	74.33564	84.164993
Feysal Kemal et al.	80.922333	75.912392	85.932274
Million Wesenu Demissie et al.	80.271233	75.025146	85.517311
Aboma Temesgen et al.	80.906898	75.905998	85.907799
Combined	80.421325	75.738724	85.10392

### 3.10 Associated Factors for Recovery From Obstetric Fistula in Ethiopia

The analysis of factors associated with time to recovery from obstetric fistula in Ethiopia identified several significant predictors amongst the 13 variables examined. Specifically, seven factors demonstrated a statistically significant relationship with recovery time. Maternal height ≤ 150 cm, attendance at antenatal care follow‐up, institutional delivery, duration of incontinence < 3 months, width fistula ≤ 2 cm, an intact urethra and vaginal delivery were all associated with a shorter time to recovery, indicating faster healing.

### 3.11 Maternal Height

**FIGURE 4:**
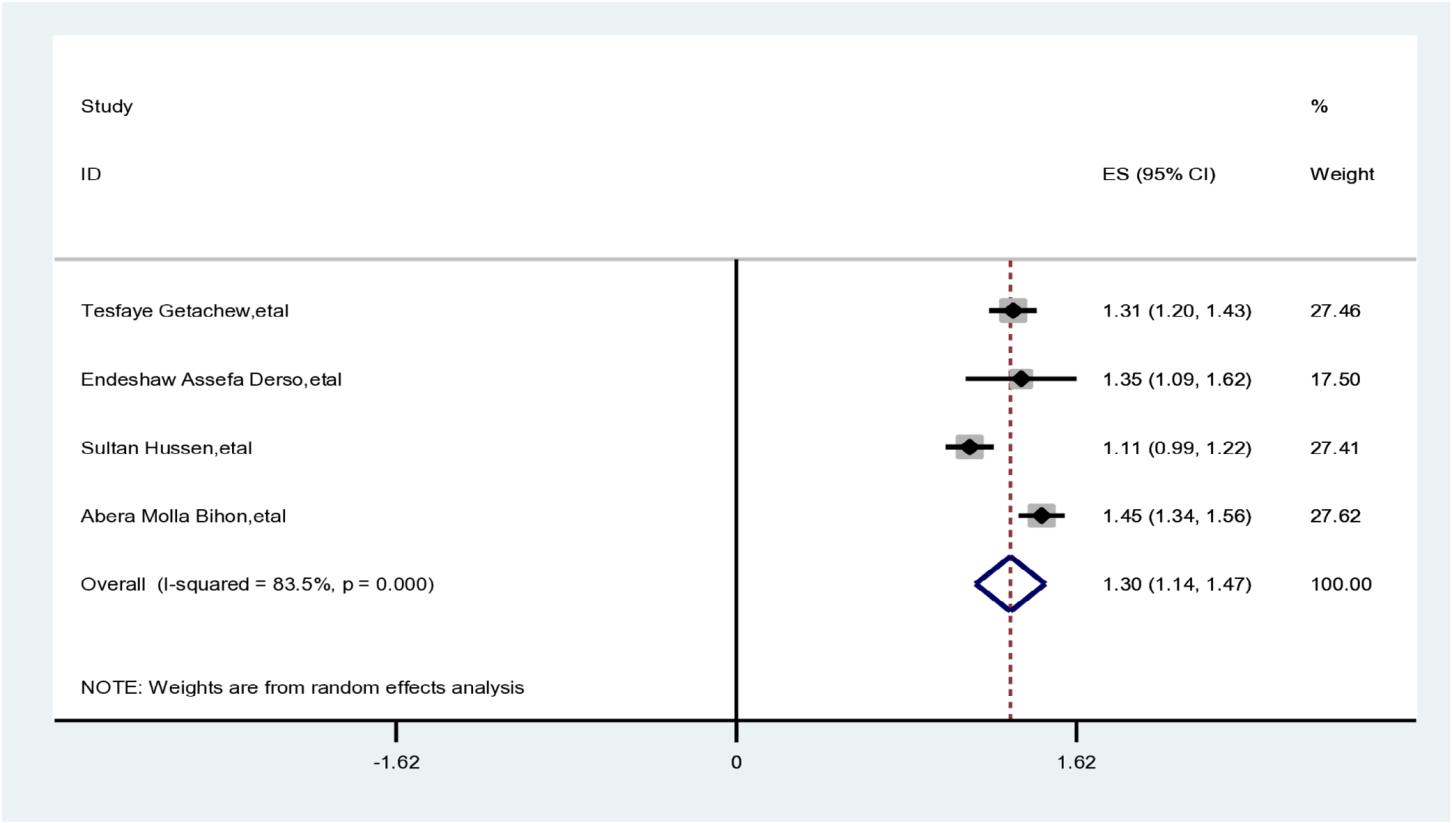
Forest plot of the pooled hazard ratio for the association between maternal height and recovery from obstetric fistula in Ethiopia.

Maternal height was significantly associated with time to recovery from obstetric fistula. Women with a height ≤ 150 cm were 1.3 times more likely to experience recovery compared to taller women (adjusted HR = 1.30, 95% CI: 1.14–1.47, *p* < 0.001; *I* ^2^ = 83.5%; Egger's test *p* = 0.937) (Figure 4). These findings suggest that women with an intact urethra tend to recover more quickly than those with urethral damage.

### 3.12 ANC Follow‐Up

**FIGURE 5:**
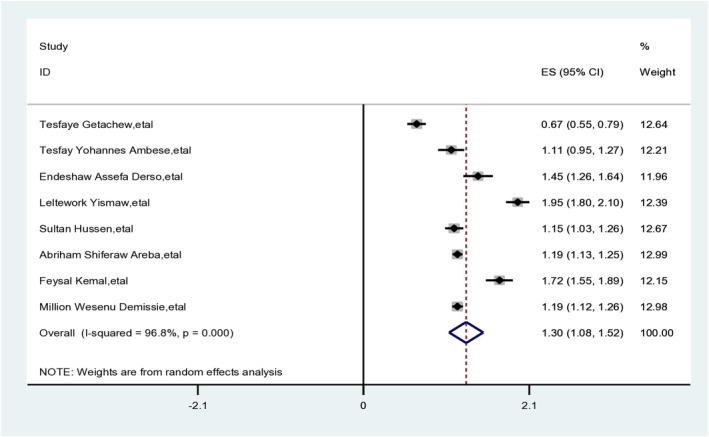
Forest plot of the pooled hazard ratio for the association between antenatal care (ANC) follow‐up and recovery from obstetric fistula in Ethiopia.

This meta‐analysis found that antenatal care (ANC) follow‐up was positively associated with recovery from obstetric fistula. Women who had ANC follow‐up were 1.3 times more likely to recover compared to those who did not (adjusted HR = 1.30, 95% CI: 1.08–1.52; *I* ^2^ = 96.8%; Egger's test *p* > 0.427) (Figure 5). This indicates that the hazard of recovery increases with ANC follow‐up.

### 3.13 Place of Delivery

**FIGURE 6:**
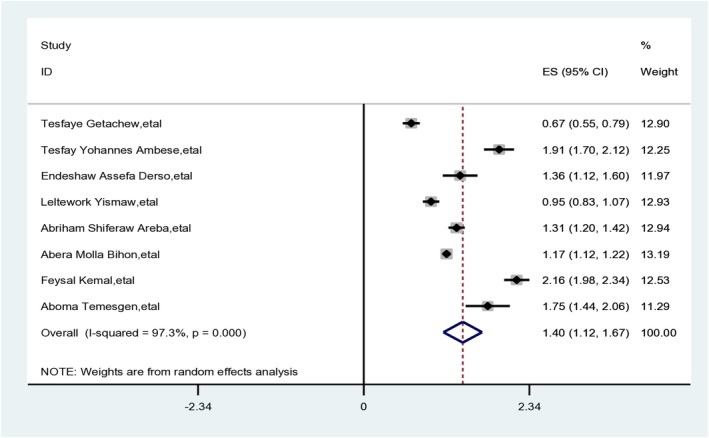
Forest plot of the pooled hazard ratio for the association between institutional delivery and recovery from obstetric fistula in Ethiopia.

This meta‐analysis found that place of delivery was positively associated with recovery from obstetric fistula. Women who delivered in a health facility were 1.4 times more likely to recover compared to those who delivered at home (adjusted HR = 1.40, 95% CI: 1.12–1.67; *I* ^2^ = 97.3%; Egger's test *p* > 0.307) (Figure 6). A random‐effects model was used due to the high heterogeneity amongst the included studies (*I* ^2^ = 97.3%, *p* < 0.001).

### 3.14 Duration of Incontinence

**FIGURE 7:**
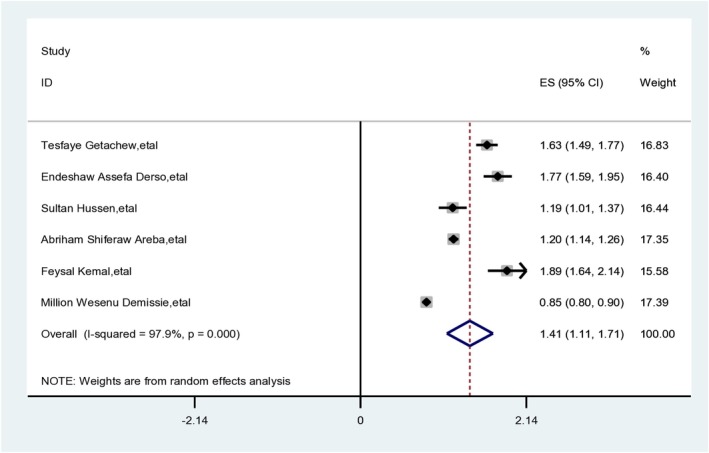
Forest plot of the pooled hazard ratio for the association between duration of incontinence and time to recovery from obstetric fistula in Ethiopia.

This meta‐analysis found that the duration of incontinence was positively associated with recovery from obstetric fistula. Women with incontinence of less than 3 months were 1.41 times more likely to recover compared to those with longer durations (adjusted HR = 1.41, 95% CI: 1.11–1.71; Egger's test *p* > 0.050) (Figure 7). A random‐effects model was applied due to significant heterogeneity amongst the included studies (I^2^ = 97.9%, *p* < 0.001).

### 3.15 Urethral Status

**FIGURE 8:**
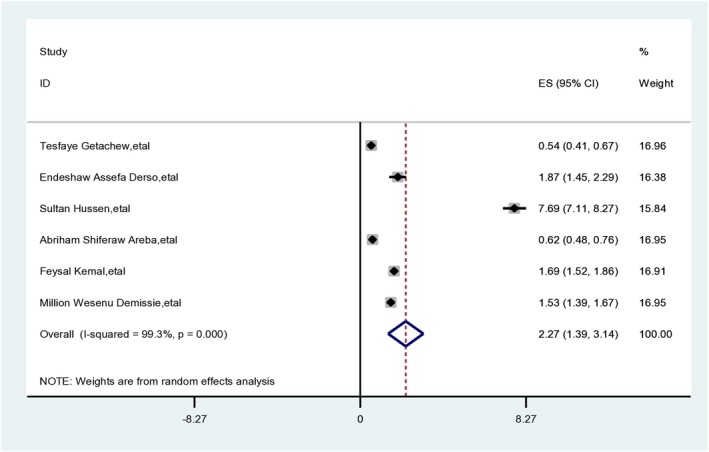
Forest plot of the pooled hazard ratio for the association between urethral status and time to recovery from obstetric fistula in Ethiopia.

The adjusted hazard of recovery for patients with an intact urethra was 2.27 times higher than for those with a damaged urethra (adjusted HR = 2.27, 95% CI: 1.39–3.14, *p* < 0.001; Egger's test = 0.063) (Figure 8). A random‐effects model was applied due to significant heterogeneity amongst the included studies (*I* ^2^ = 99.3%, *p* < 0.001).

### 3.16 Mode of Delivery

**FIGURE 9:**
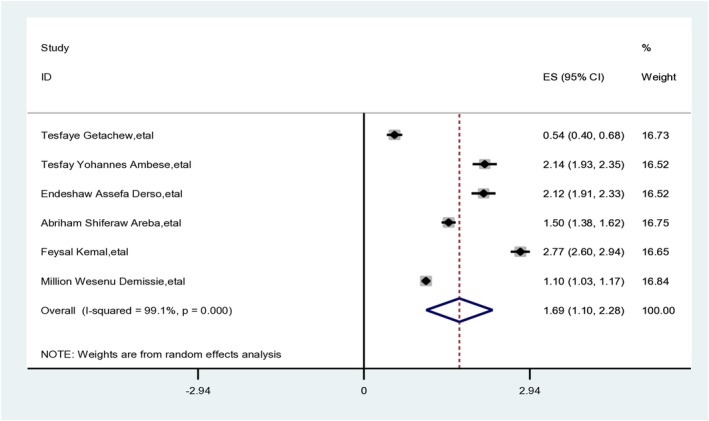
Forest plot of the pooled hazard ratio for the association between vaginal delivery and recovery from obstetric fistula in Ethiopia.

This meta‐analysis found that mode of delivery was positively associated with recovery from obstetric fistula. Women who delivered vaginally had a 1.69 times higher hazard of recovery compared to those who delivered by caesarean section (adjusted HR = 1.69, 95% CI: 1.10–2.28; Egger's test *p* > 0.176) (Figure 9). A random‐effects model was used due to significant heterogeneity amongst the included studies (*I* ^2^ = 99.1%, *p* < 0.001).

#### 3.16.1 Width of Fistulae

**FIGURE 10:**
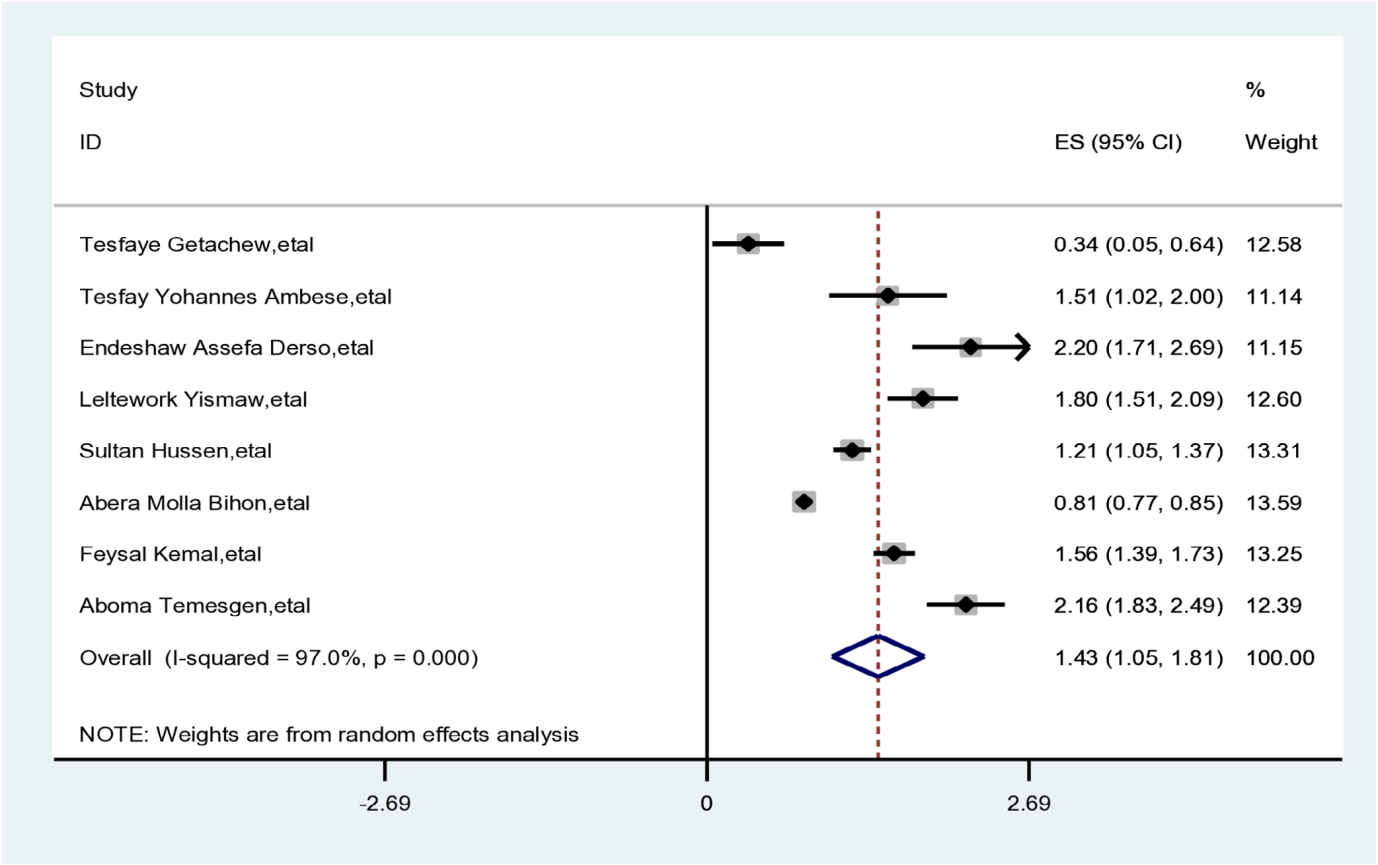
Forest plot of the pooled hazard ratio for the association between fistula width and recovery from obstetric fistula in Ethiopia.

This meta‐analysis found that fistula size was positively associated with recovery from obstetric fistula. Women with a fistula width ≤ 2 cm had a 1.43 times higher hazard of recovery compared to those with a fistula > 2 cm (adjusted HR = 1.43, 95% CI: 1.05–1.81; Egger's test *p* = 0.031) (Figure 10). A random‐effects model was applied due to significant heterogeneity amongst the included studies (I^2^ = 97.0%, *p* < 0.001).

### 3.17 Factors Not Significantly Associated With Recovery From Obstetric Fistula

Amongst the variables included in this analysis, maternal age, weight, duration of labour, fistula length, residence and education were not significantly associated with recovery from obstetric fistula.

### 3.18 Maternal Age

**FIGURE 11:**
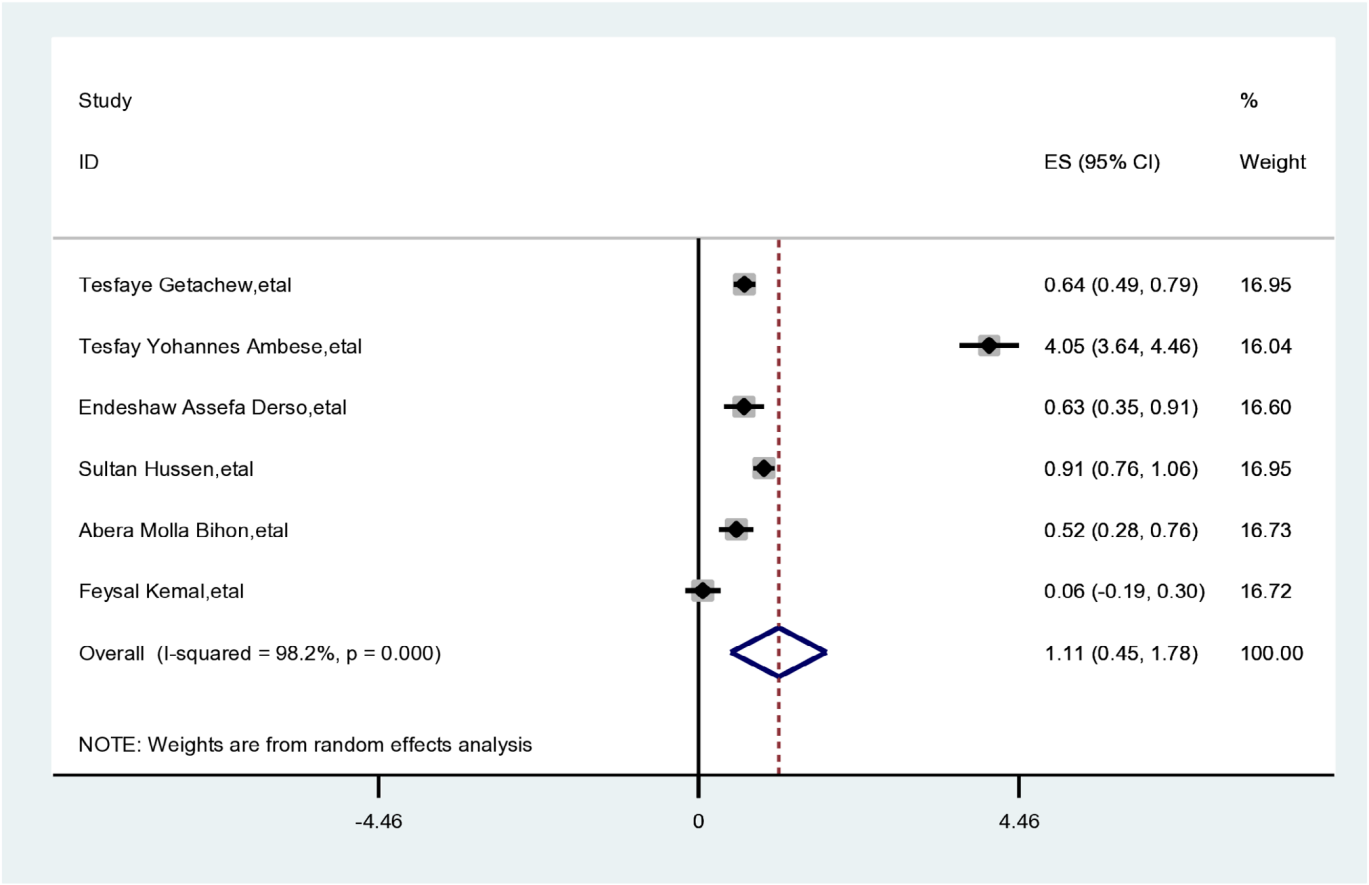
Forest plot of the pooled hazard ratio for the association between maternal age and recovery from obstetric fistula in Ethiopia.

Maternal age < 18 years was not significantly associated with recovery time from obstetric fistula (adjusted HR = 1.11, 95% CI: 0.45–1.78; Egger's test = 0.341) (Figure 11). A random‐effects model was applied due to significant heterogeneity amongst the included studies (*I* ^2^ = 98.2%, *p* < 0.001).

### 3.19 Maternal Weight

**FIGURE 12:**
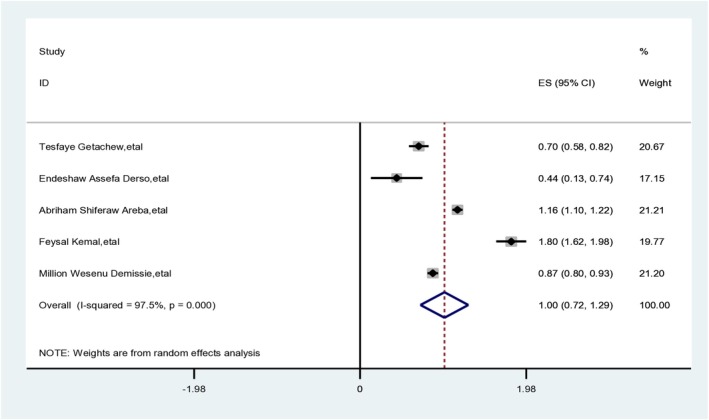
Forest plot of the pooled hazard ratio for the association between maternal weight and time to recovery from obstetric fistula in Ethiopia.

Maternal weight < 50 kg was not significantly associated with recovery from obstetric fistula (adjusted HR = 1.00, 95% CI: 0.72–1.29; Egger's test = 0.978) (Figure 12). A random‐effects model was applied due to significant heterogeneity amongst the included studies (*I* ^2^ = 97.5%, *p* < 0.001).

### 3.20 Duration of Labor

**FIGURE 13:**
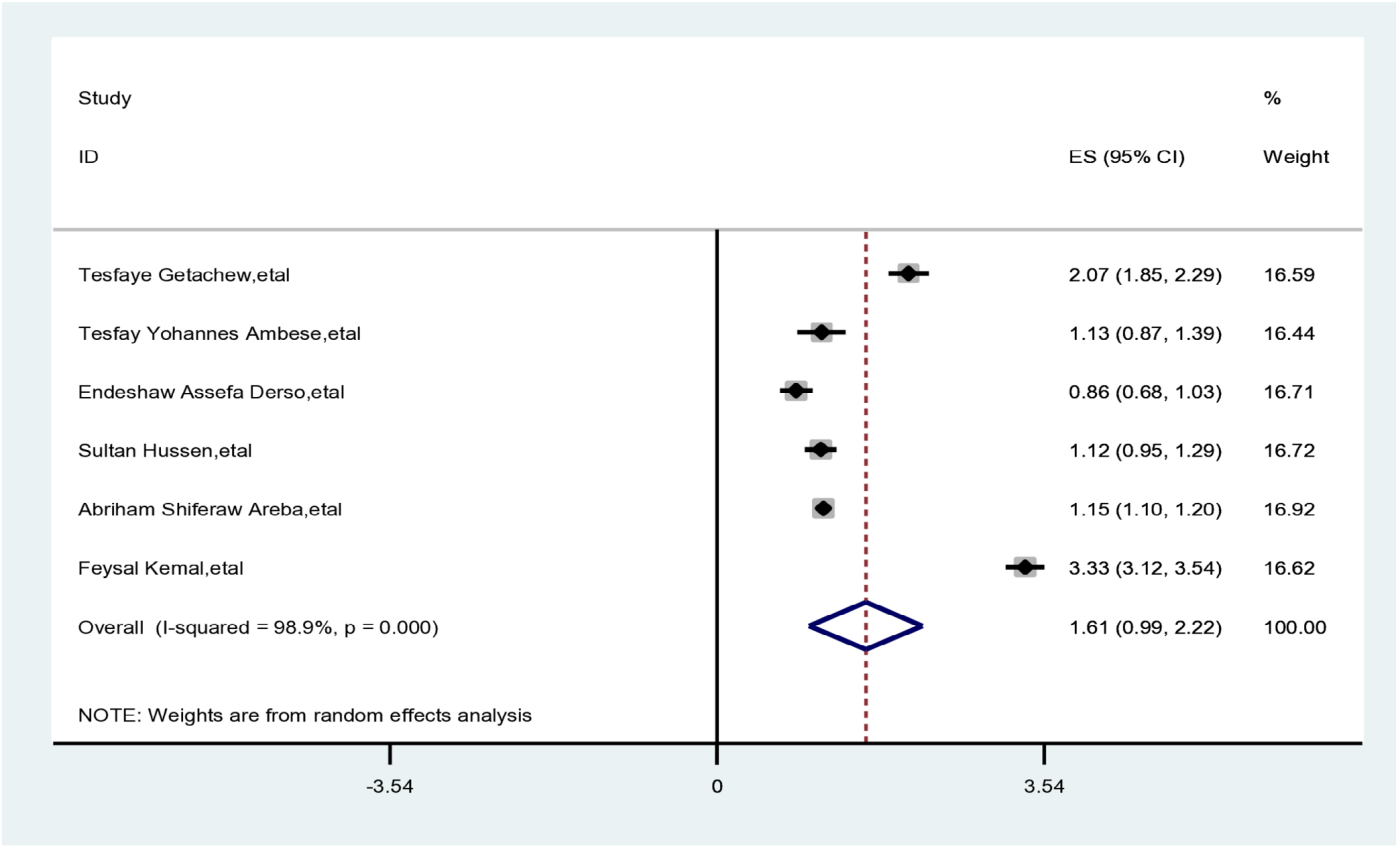
Forest plot of the pooled hazard ratio for the association between duration of labour and time to recovery from obstetric fistula in Ethiopia.

Labour duration of less than 2 days was not significantly associated with recovery from obstetric fistula (adjusted HR = 1.61, 95% CI: 0.99–2.22; Egger's test = 0.350) (Figure 13). A random‐effects model was applied due to significant heterogeneity amongst the included studies (*I* ^2^ = 99.8%, *p* < 0.001).

### 3.21 Length of the Fistula

**FIGURE 14:**
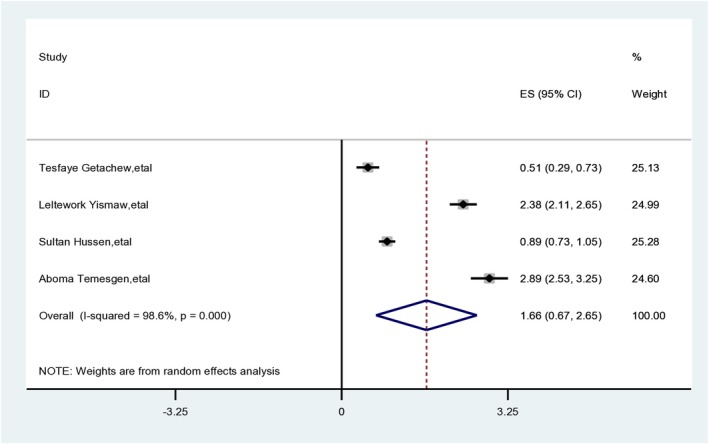
Forest plot of the pooled adjusted hazard ratio for the association between fistula length and time to recovery from obstetric fistula in Ethiopia.

Fistula length was not significantly associated with recovery time from obstetric fistula (adjusted HR = 1.66, 95% CI: 0.67–2.65; Egger's test = 0.208) (Figure 14). A random‐effects model was applied due to substantial heterogeneity amongst the included studies (*I* ^2^ = 98.6%, *p* < 0.001).

### 3.22 Residence

**FIGURE 15:**
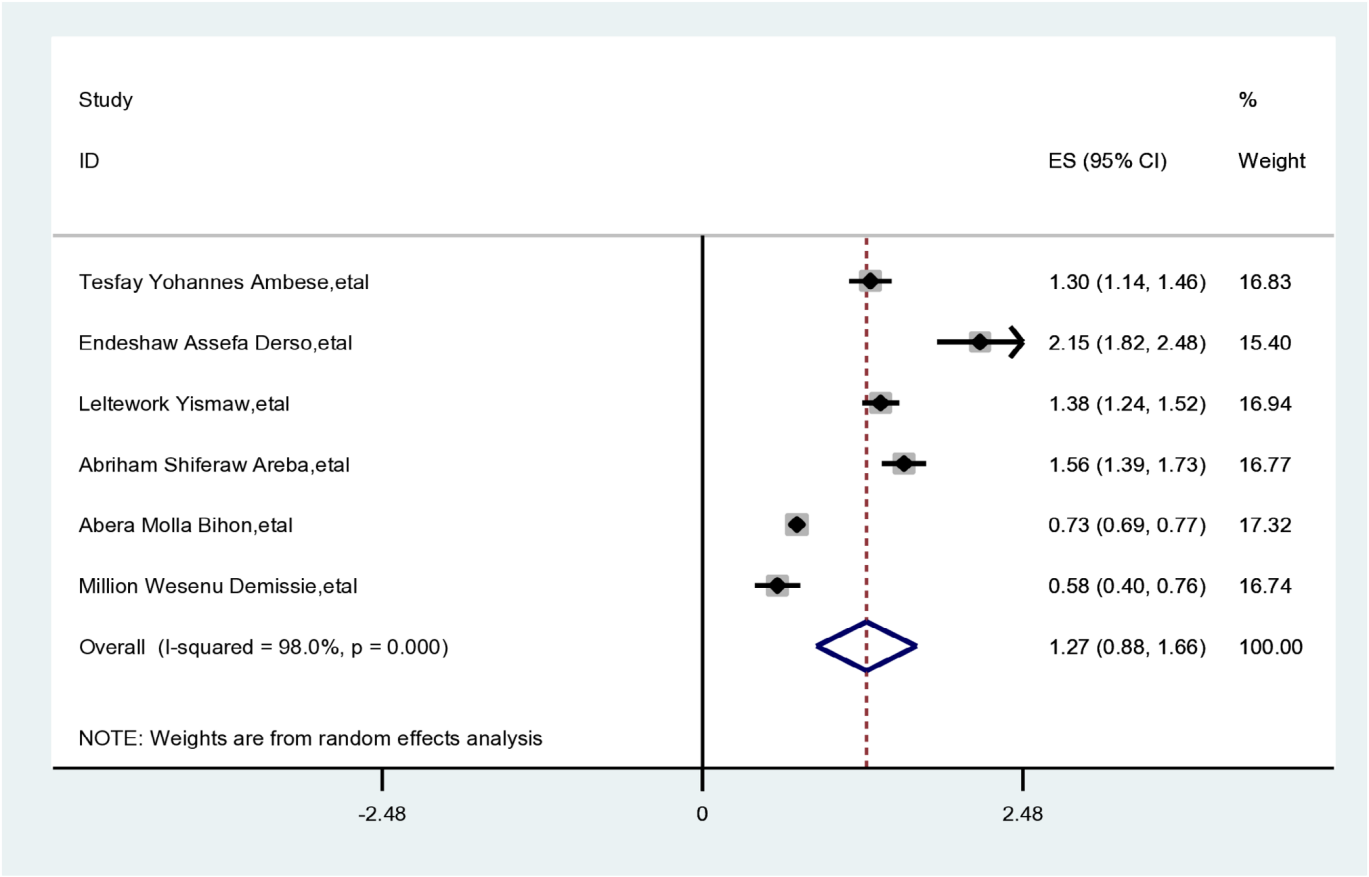
Forest plot of the pooled hazard ratio for the association between urban residence and time to recovery from obstetric fistula in Ethiopia.

Urban residence was not significantly associated with recovery from obstetric fistula (adjusted HR = 1.27, 95% CI: 0.88–1.66; Egger's test = 0.048) (Figure 15). A random‐effects model was applied due to substantial heterogeneity amongst the included studies (*I* ^2^ = 98%, *p* < 0.001).

### 3.23 Maternal Education

**FIGURE 16:**
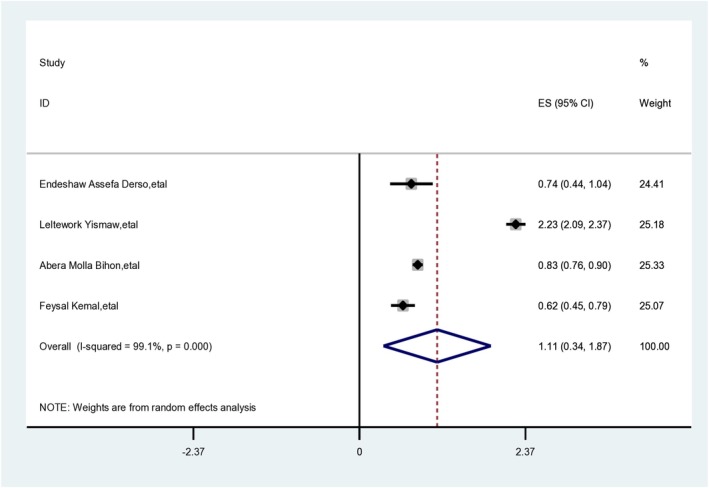
Forest plot of the pooled hazard ratio for the association between maternal education and time to recovery from obstetric fistula in Ethiopia.

Literacy was not significantly associated with recovery from obstetric fistula (adjusted HR = 1.11, 95% CI: 0.34–1.84; Egger's test = 0.794) (Figure 16). A random‐effects model was applied due to substantial heterogeneity amongst the included studies (*I* ^2^ = 99.1%, *p* < 0.001).

## 4 Discussion

Obstetric fistula remains a major public health concern in Ethiopia and sub‐Saharan Africa, and recovery from this condition is critical for improving affected women's quality of life (UNFPA 2012). In this meta‐analysis, the overall pooled prevalence of recovery from obstetric fistula in Ethiopia was 80.42% (95% CI: 75.74–85.10), indicating that a substantial proportion of women achieve successful repair. Several predictors of recovery were identified, including maternal height < 150 cm, institutional delivery, vaginal delivery, duration of incontinence ≤ 3 Months, fistula width ≤ 2 cm, intact urethra and having antenatal care (ANC) follow‐up.

Our finding that shorter women (< 150 cm) had a 1.3‐fold higher likelihood of recovery contrasts with previous research (Gezimu 2023) and appears counterintuitive. This paradoxical result may reflect the complex interaction between childhood nutrition, physical development and obstetric outcomes. Short stature often results from early‐life malnutrition and stunting, which are associated with an increased risk of cephalopelvic disproportion during childbirth—a recognised risk factor for obstetric fistula. However, it is possible that shorter women in this study presented with fistulas involving less extensive tissue damage or sought care earlier, potentially due to heightened clinical attention, facilitating more successful surgical repair. This finding should be interpreted cautiously, as it may be influenced by unmeasured confounders such as nutritional status, type of fistula or differences in care pathways, and requires further research for confirmation.

Women with smaller fistula widths (< 2 cm) were 1.43 times more likely to recover, consistent with previous studies (Moghimi‐Dehkordi et al. 2008; Cox 2018). This finding can be explained by several factors related to the nature of smaller fistulas. First, small‐sized fistulas involve less tissue damage, with minimal necrosis and reduced inflammation, making the surgical repair technically easier. The edges of the fistula are often healthier and more amenable to precise suturing, allowing for better approximation of the wound and more efficient healing. Additionally, smaller fistulas require less extensive dissection, reducing operative time and the risk of postoperative complications, which collectively contribute to faster recovery. These factors highlight why fistula size is a critical predictor of successful surgical outcomes (Sjøveian et al. 2011).

It was also revealed that women with intact urethral damage were 1.39 times more likely to recover from obstetric fistula compared to women with damaged urethra. This is supported by previous literature (Ockrim et al. 2009; Wall et al. 2005). The possible reason might be because the urethra is usually fixed to the pubic bone, making it difficult to move the urethra's length and the damaged urethra becomes de‐enervated and shortened, which makes it difficult to anastomose a detached urethra (Barone et al. 2012; Tebeu Marie et al. 2019). Similar findings were reported in a systematic review from sub‐Saharan Africa, which identified urethral damage as a predictor of repair failure and delayed recovery (Frajzyngier, Ruminjo, and Barone 2012; Hareru et al. 2024).

Institutional delivery was associated with a 1.41‐fold higher recovery rate compared to home delivery. This association can be explained by the quality and safety of care provided in health facilities. Institutional deliveries typically follow aseptic techniques, involve skilled birth attendants, and minimise unnecessary vaginal or instrumental manipulation, reducing the risk of additional trauma to the urogenital tract. In contrast, home deliveries often occur without sterile equipment or skilled assistance, and repeated manipulations during prolonged labour can exacerbate tissue injury, leading to larger or more complex fistulas. Furthermore, women delivering in health facilities have better access to timely obstetric interventions and early detection of complications, which likely contributes to faster and more successful recovery from obstetric fistula (Haroun et al. 2001).

The hazard rate of incontinence ≤ 3 months was 1.41 times recovery from obestetric fistula than that of > 3 months incontinence. This is primarily because prolonged leakage of urine or faeces irritates and inflames the surrounding tissues, increasing the risk of infection and tissue damage. Over time, chronic exposure can lead to fibrosis and scarring, making surgical repair more difficult and slowing the healing process. In contrast, recently developed fistulas have cleaner tissue edges, minimal necrosis and less contamination, which facilitates easier surgical closure and faster recovery (Frajzyngier, Ruminjo, and Barone 2012). Additionally, women with shorter‐duration incontinence are less likely to experience complications such as recurrent infections or poor general health, further supporting a more rapid and successful recovery.

Moreover, in this study, we found that vaginal delivery was associated with a 1.69‐fold higher likelihood of recovery from obstetric fistula compared to caesarean delivery. This finding may be justified by the fact that vaginal deliveries generally involve less surgical manipulation and lower risk of infection or trauma to adjacent pelvic structures, which can facilitate faster tissue healing and reduce postoperative complications. In contrast, caesarean deliveries may increase the risk of uterine or pelvic infections and additional tissue injury, potentially prolonging recovery. These results are consistent with previous studies reporting that less invasive delivery methods are associated with more favourable outcomes following fistula repair (Jabbar et al. 2016; Jonas and Petri 1984; Lee et al. 1988).

The recovery time of obstetric fistula patients who had a history of antenatal care (ANC) follow‐up was 1.3 times shorter than that of patients without ANC follow‐up. This finding can be justified by the role of ANC in providing general counselling and health education, which increases women's awareness of pregnancy‐related risks and danger signs during labour and delivery. Consequently, women who attend ANC are more likely to seek timely medical care and present earlier for fistula treatment, facilitating prompt surgical intervention and faster recovery. Additionally, regular ANC visits may improve women's overall health status and preparedness for childbirth, further contributing to more favourable recovery outcomes (Amini 2000).

The observed variability in recovery outcomes across studies may be influenced by differences in surgical techniques, surgeon expertise and postoperative care protocols. Given that the included studies spanned a long period and multiple regions of Ethiopia, it is likely that these contextual factors varied between centres. Variations in surgical approaches, experience of the operating surgeons and adherence to post‐operative care standards including catheterization duration, infection prevention, and follow‐up practises, could contribute to differences in recovery rates. Recognising these sources of variability is important for interpreting the pooled estimates and highlights the need for standardised clinical protocols to optimise fistula repair outcomes across different settings.

Future research should consider qualitative approaches, as they can provide in‐depth insights into women's lived experiences, uncover cultural and social factors influencing recovery and inform patient‐centred interventions and policies that may not be fully captured through quantitative studies.

### 4.1 Limitations

This study has limitations that should be considered when interpreting the findings. First, all included studies were retrospective cohort designs conducted exclusively in Ethiopia, which may limit the generalizability of the results to other countries or settings with different healthcare systems, cultural practises or population characteristics. Second, there was a high level of statistical heterogeneity across studies (*I* ^2^ values ranging from 83.5% to 99.3%), which may affect the precision of the pooled estimates and the comparability of results. Although sensitivity analyses, subgroup analyses, meta‐regression and Trim and Fill analyses were conducted to assess the robustness of the findings. Third, the definition of recovery of obstetric fistula hospital discharge after surgery, and differences in discharge criteria across centres, could have introduced bias in outcome assessment. Despite these limitations, this systematic review and meta‐analysis provide valuable insights into the prevalence of recovery from obstetric fistula and its associated factors, offering guidance for targeted interventions and improving maternal health outcomes in Ethiopia.

## 5 Conclusion

The pooled prevalence of recovery from obstetric fistula was 79.23%. Factors significantly associated with recovery included maternal height, institutional delivery, mode of delivery, duration of incontinence ≤ 3 months, width of fistula ≤ 2 cm, intact urethra and antenatal care follow‐up. Based on these findings, targeted interventions are recommended: increasing ANC uptake, promoting institutional deliveries and ensuring early management of incontinence to improve recovery outcomes. It is equally important to provide equitable and accessible basic obstetric care at the community level, comprehensive emergency obstetric care at referral centres and timely treatment for women and adolescent girls living with fistula. Strengthening political and financial commitment, translating policies into actionable programmes and incorporating fistula and maternal morbidity into national health plans and budgets are critical steps towards sustainable improvements in maternal health.

## Author Contributions

All authors contributed to the study protocol and C.M. wrote the methodology, conceptualization and draught of the manuscript. **H.K**. and **G.K.:** supervision and formal analysis. **T.E**. and **A.S.:** final draught of manuscript and methodology. **A.A**., **A.Y**., and **E.M.:** methodology, conceptualization and investigation.

## Funding

The authors have nothing to report.

## Ethics Statement

This study is a systematic review and meta‐analysis that did not involve the collection of primary data from human or animal subjects.

## Consent

There are no human participants in this article, and informed consent is not required.

## Conflicts of Interest

The authors declare no conflicts of interest.

## Supporting information


**Table S1:** shows Newcastle–Ottawa Scale score of the included studies of recovery of obstetric fistula patients and factors in Etiopia.

**File S1:** nop270680‐sup‐0002‐FileS1.docx. PRISMA 2020 checklist.

## Data Availability

The data that supports the findings of this study are available in the Supporting Information of this article.
