# Supplementary material for: Determinants of Recovery From Obstetric Fistula in Ethiopia: A Systematic Review and Meta‐Analysis
Source: Nurs Open. 2026 Jul 6;13(7):e70680. doi: 10.1002/nop2.70680 (PMC13335612; doi:10.1002/nop2.70680)
Supplement: Supplementary file 1 — Table S1: shows Newcastle–Ottawa Scale score of the included studies of recovery of obstetric fistula patients and factors in Etiopia. [file NOP2-13-e70680-s002.docx]

Supplemental Table 1: shows Newcastle-Ottawa Scale score of the included studies of recovery of obstetric fistula patients and factors in Etiopia.

| Author | **Selection** | | | | **Comparability** | **Outcome** | | **Total** |
| --- | --- | --- | --- | --- | --- | --- | --- | --- |
|  | Representativeness s (1) | Sample size(1) | Non respondents (1) | Rrisk factor (2) | Comparable, based on the study design or analysis. confounding factors are controlled (1) | Assessment of the outcome (2) | Stastical test(1) |  |
| Tesfaye Getachew,et al. | 1 | 1 | 1 | 1 | 0 | 1 | 1 | 7 |
| Tesfay Yohannes Ambese,et al. | 1 | 1 | 0 | 1 | 1 | 1 | 1 | 6 |
| Endeshaw Assefa Derso,et al. | 1 | 1 | 1 | 1 | 1 | 1 | 1 | 7 |
| Leltework Yismaw,et al. | 1 | 1 | 1 | 2 | 1 | 1 | 1 | 8 |
| Sultan Hussen,et al. | 1 | 0 | 1 | 1 | 1 | 1 | 1 | 8 |
| Abriham Shiferaw Areba,et al. | 1 | 1 | 0 | 2 | 1 | 1 | 1 | 9 |
| Abera MollaBihon,et al. | 1 | 1 | 1 | 1 | 1 | 1 | 1 | 7 |
| Feysal Kemal,et al. | 1 | 1 | 1 | 2 | 1 | 1 | 1 | 8 |
| Million Wesenu Demissie,et al. | 1 | 1 | 1 | 1 | 1 | 1 | 1 | 7 |
| Aboma Temesgen,et al. | 1 | 1 | 1 | 2 | 1 | 1 | 1 | 8 |

**Very Good Studies: 9 points**

**Good Studies: 7-8 points**

**Satisfactory Studies: 5-6 points**

**Unsatisfactory Studies: 0 to 4 points**
